# Search for an Appropriate Behavior within the Emotional Regulation in Virtual Creatures Using a Learning Classifier System

**DOI:** 10.1155/2017/5204083

**Published:** 2017-10-25

**Authors:** Jonathan-Hernando Rosales, Félix Ramos, Marco Ramos, José-Antonio Cervantes

**Affiliations:** ^1^Department of Computer Science, Cinvestav-IPN, Unidad Guadalajara, Av. del Bosque No. 1145, 45019 Zapopán, JAL, Mexico; ^2^Department of Computer Science, Universidad Autónoma del Estado de México, Instituto Literario, No. 100, 50000 Toluca, MEX, Mexico; ^3^Department of Computer Science and Engineering, Los Valles University Center, University of Guadalajara, Carretera Guadalajara-Ameca Km. 45.5, Ameca, JAL, Mexico

## Abstract

Emotion regulation is a process by which human beings control emotional behaviors. From neuroscientific evidence, this mechanism is the product of conscious or unconscious processes. In particular, the mechanism generated by a conscious process needs a priori components to be computed. The behaviors generated by previous experiences are among these components. These behaviors need to be adapted to fulfill the objectives in a specific situation. The problem we address is how to endow virtual creatures with emotion regulation in order to compute an appropriate behavior in a specific emotional situation. This problem is clearly important and we have not identified ways to solve this problem in the current literature. In our proposal, we show a way to generate the appropriate behavior in an emotional situation using a learning classifier system (LCS). We illustrate the function of our proposal in unknown and known situations by means of two case studies. Our results demonstrate that it is possible to converge to the appropriate behavior even in the first case; that is, when the system does not have previous experiences and in situations where some previous information is available our proposal proves to be a very powerful tool.

## 1. Introduction

The need for virtual creatures with behaviors similar to human beings has been increasing in recent years in different areas, for instance, disaster simulation, serious games, and training, because of the need for increasing interaction between human beings and virtual environments. The creation of these virtual creatures is an open problem due to the complexity involved. It is evident that emotions play a key role in human behavior; current research uses different approaches to deal with the problem of computing behavior for virtual creatures while taking emotions into account.

**Figure 1:**
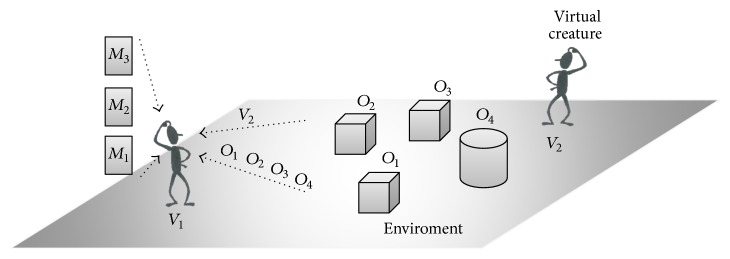
Environment. Environment example for virtual creatures, showing a possible environment for virtual creatures where *O*_1_,…, *O*_*n*_ are possible objects perceived, *V*_1_,…, *V*_*n*_ are other virtual creatures perceived, and *M*_1_,…, *M*_*n*_ are possible memories recovered from previous situations.

A human being's emotion is the psychophysiological result of the process of perceiving stimuli. A stimulus is a set of objects, situations, or memories with which the human being interacts within the environment [1] (see Figure 1). The emotion is made up of an internal emotional state and an emotional behavior response to the environment. When it comes to the development of emotions, there are emotional theories about how human beings carry out an emotional assessment of the environment in order to generate emotions from a stimulus or a set of stimuli within a situation. In particular, we use the appraisal theory [2], because it presents a general process for emotions. Also, it is widely cited and used in research studies making up the state of the art. Appraisal theory is a psychological theory that considers emotions as appraisals of the environment. Gross's approach (an extension of appraisal theory) considers three appraisals to compute the emotion process.

- The first appraisal gives a certain emotional value to a perceived stimulus in order to differentiate it from the rest of the stimuli perceived in the environment. A stimulus can have different appraisals depending on the perceived situation.
- The second appraisal helps to understand the individual involvement in the situation. At this step, the sum of stimuli perceived in the environment generates the emotional state. This emotional state generates an emotional behavior (fear, anger, joy, etc.) in response to the perceived stimulus. Humans generate multiple appraisals for a specific situation, due to the diversity of appraisals that each perceived stimulus has in that situation.
- The third appraisal helps to choose what to do in response to the specific situation. In contrast to the previous appraisals, this appraisal computes multiple responses to a specific situation perceived in the environment. Thus, in this case, a process is needed to choose the most appropriate response to the current situation (a decision-making process).

However, in order to compute behavior for virtual creatures it is not enough to take emotions into account. For instance, there are situations where emotions must be regulated in order to reach an objective. In this case, the third appraisal must consider the emotion regulation needed to reach an objective. In other words, if the computed emotion does not contribute to reaching the objective, the emotion regulation process helps to modify the emotional behavior in order to reach the objective [3].

An emotion regulation mechanism is a bridge between emotion and cognition in the neurosciences. In human beings, it helps to achieve objectives through a cognitive change [1, 4, 5]. Appropriate behavior is defined in this paper as the behavior that best serves a virtual creature's objective to achieve the maximum possible reward in a given situation. Emotion regulation is achieved by modifying the virtual creature's body and facial expressions and/or by modifying the cognitive meaning of the objects within the setting in order to direct the behavior [4]. For instance, if the leader of a project in a company is angry with a key worker, his emotions might suggest to him firing the worker. However, the leader must regulate his emotions to keep him from firing the key resource, thus ensuring the successful completion of the project; otherwise, the project will not be completed satisfactorily. This example shows the importance of the emotion regulation process.

Emotion regulation can be the result of conscious or unconscious processes. Unconscious emotion regulation is carried out by changing the stimulus perceived in the environment. By changing the perceived emotional stimulus, the emotion generation process restarts and the internal emotional state changes, thus generating a different response behavior to the situation. The conscious emotional regulation process, on the other hand, is carried out using emotional regulation techniques [4]. These techniques can be classified into two groups: emotional regulation achieved by changing the meaning of the perceived stimulus and emotional regulation achieved by suppressing the response behavior. These two techniques require a prior mechanism for their functioning. This prior mechanism is responsible for identifying what behavior would be appropriate to meet a specific objective, as in the previous example where the project leader needs to control his behavior to avoid aborting a project. The appropriate behavior is generated from previous experiences when they are available [3], the conclusion being that learning is the prior mechanism required to determine appropriate behavior for a given situation in the environment. Thus, information for emotional regulation is given by the fundamental process of memory.

In this article, we propose a mechanism for the automatic generation of appropriate behavior in a situation that requires emotion regulation. The structure of this article is as follows: Section 2 presents some work done in the field of cognitive architectures dealing with emotions and the search for appropriate behaviors.  Section 3 presents the theoretical evidence of emotion regulation as well as neuroscientific evidence related to the search for appropriate behavior within the brain.  Section 4 presents our proposal for a virtual creature finding an appropriate behavior for a specific situation.  Section 5 presents a case study that showcases the functionality of the proposal. Finally, conclusions and a discussion of the results are presented.

## 2. Related Work

There are a number of studies that propose emotional models [7–10], among other cognitive processes that are inspired by biological evidence. Some of the most important proposals for this work deal with cognitive architectures that include multiple cognitive processes working together to compute a human-like behavior to be exhibited by virtual creatures. These cognitive architectures for virtual creatures are inspired mainly by psychological theories and/or neuroscientific evidence.

Some of the existing cognitive architectures that consider emotion in the computation of behavior are presented below. The selection is based on the theory of emotion assessment. We look at the implementation of all steps of the appraisal theory. In particular, we want to see the possibility of regulating emotion consciously in these processes.

### 2.1. SOAR

State Operator and Result, “SOAR” [7], is a cognitive architecture for artificial intelligence. It is used for the development of intelligent agents that solve problems ranging from simple to complex open problems. The design of SOAR is based on the assumption that all deliberative behavior-oriented goals can be formulated as the selection of operators and their application to the state.

Thus, if an agent has a particular goal in a specific situation, this goal can be achieved by different actions and in multiple ways. The state is a representation of the current situation. The possible actions in such specific situations are the possible operations for that state.

SOAR has not fully implemented the emotional process. However, it proposes implementing the generation of several hypotheses based on appraisal theory. This architecture uses the three steps of appraisal theory; however, it does not implement these steps and reduces deployment to a simple tool for improving the virtual creature's learning through a greater reward function when it selects a good action in a specific scene. SOAR does not contemplate different meanings of the perceived stimulus.

Given the absence of a full emotional evaluation process, the agent may not compute an appropriate emotional behavior if it does not consider multiple cognitive meanings of the same stimulus within the environment, or an emotional state of the virtual creature within their case studies.

### 2.2. iCub

Integrated Cognitive Universal Body, “iCub” [9], is a cognitive architecture designed for virtual creatures and humanoid robots. This architecture seeks to copy cognitive processes in humans and incorporate them into the humanoid robots. The approach is based on psychological and neuroscientific evidence. The architecture is implemented in the iCub Humanoid robot, which has the appearance of a 2.5-year-old child, and the objective is to provide it with the basic skills that a boy of that age possesses. The architecture is not yet complete; it is only a preliminary architecture.

Through three components, iCub generates an affective state. This affective state is equal to the emotional state in appraisal theory. These components are curiosity (dominated by external stimuli), experimentation (dominated by internal stimuli), and social commitment (based on a good balance between external and internal stimuli). These three components generate the affective state in conjunction with a process of selection of action that generates a small homeostatic process that regulates the iCub robot's behavior. iCub generates simple basic emotions such as joy, fear, anger, and sadness. It also generates behavior that is similar to a little child's. The emotional process is simple and focuses on the interaction with the environment, ignoring conscious emotional processes.

Emotions in iCub are regulated by the change of stimuli in the setting. That is, it does not have a process of conscious emotion regulation.

### 2.3. Kismet

Kismet [10–12], developed by MIT (Massachusetts Institute of Technology), is capable of expressing emotions; it was developed in the 1990s. The architecture developed for this robot began as a working framework made up of four subsystems:

- Motivation system, which consists of handlers and emotions
- Behavior system, which consists of several types of behavior
- System of perception and attention, which extracts the characteristics from the atmosphere
- Motor system, which runs facial expressions.

There are seven emotions expressed in Kismet, based on the theory of basic emotions [6]. The emotions can be used for three purposes: first, to influence the behavior of the robot by giving preference to one behavior over others; second, to have an impact on the robot's emotional state, which in turn will be shown through the motor system; and third, to serve as a learning mechanism: after the completion of predetermined satisfactions, the robot can learn the way it accomplished the task or not. Facial expressions are predefined to produce a motor response commensurate to the emotions. As in iCub, Kismet regulates its emotions in response to changes in the environment. It does not have conscious emotion regulation.

## 3. Theoretical Evidence

In Section 2 we described some cognitive architectures. From their descriptions, it is possible to see why they are unable to compute an appropriate natural behavior in certain situations. This comes from not taking into account factors such as conscious emotion regulation that can influence the achievement of objectives within the environment.

There is biological evidence that shows how human beings seek an appropriate behavior when they face situations [3, 13–15]. In order to compute the appropriate behavior, humans take into account prior knowledge regarding similar situations and the association of known situations with the current situation (known or unknown). However, it is also true that emotions can make humans' behavior inappropriate in specific situations. In those cases, emotion regulation is needed. Emotion regulation consists of trying to ignore stimuli that cause emotional overflow or suppressing the emotional behavior, that is, pretending that we are not feeling the emotion. But how do human beings do this? And where in the brain are these processes carried out?

Human beings associate old knowledge with new. This is done by identifying similar characteristics between known and unknown situations. This process is carried out within the brain in areas that retrieve information and high-level cognitive processes.

Table 1 summarizes neuroscientific evidence showing the areas involved in emotional regulation. Some of these are the Dorsomedial Prefrontal Cortex (DMPFC), the Dorsolateral Prefrontal Cortex (DLPFC), the Ventrolateral Prefrontal Cortex (VLPFC), the Orbitofrontal Cortex (OFC), the Amygdala (AMYGDALA), the Hippocampus (HIPPOCAMPUS), the Insula (INSULA), the Ventral Striatum (VS), the rostral Anterior Cingulate Cortex (rACC), and the subgenual Anterior Cingulate Cortex (sgACC) [16–21]. As described previously, our proposal is based on neuroscientific evidence. Thus, these areas are taken as the basis for the development of our model of emotion regulation for virtual creatures. Reference [3] defines a flow of information between these areas based on neuroscientific evidence and also on the model proposed by Gross for emotion regulation [22–25].

The elements of our proposed architecture are explained below, including a few elements to complete the information flow.

*The Dorsomedial Prefrontal Cortex (DMPFC)* is associated with the choice of behavior [15]. This area of the brain is active during the identification of situations. It associates small parts of the setting with some situation previously stored in the brain. Thus, it generates a behavior according to the information stored from previous similar lived situations. It is even possible to predict social consequences from stored information [13–15, 26]. Although this area ensures the generation of consistent behavior, it does not mean that the proposed behavior is the most appropriate. However, it is a good beginning for learning in an unknown situation.

In our model, this structure is responsible for choosing an appropriate emotional behavior from the available options. It is fed with recollections or memories, which are received from the DLPFC. If the appropriate behavior requires a different emotional state, it is sent to the VLPFC. Finally, the computed behavior is sent to the sgACC, where a deletion process takes place [27, 28], if required.

*The Dorsolateral Prefrontal Cortex (DLPFC)* is believed to include participation in the working memory, the preparation of the response and the selection of the response [19, 29]. This structure is associated with the recovery of information and is one of the structures that allow access to the working memory within the frontal lobe [29]. It is associated with the selection of the response, probably due to its proximity to structures dedicated to executive planning.

We use this structure to access the working memory in order to generate a set of plans. The decision-making cognitive function will select one plan from this set in a given situation.

*The Ventrolateral Prefrontal Cortex (VLPFC)* is associated with the suppression of emotional responses in a changing situation [16, 17, 19]. This area decreases the emotional response through a cognitive process, which involves changing the meaning of the scene to reinterpret the situation [17, 18].

In our model, this structure reassesses the incoming stimuli, trying to give them another cognitive meaning in order to achieve a better fluidity of the desired emotional behavior. It receives the memories provided by the DLPFC and sends its results to the Amygdala in order to have a second emotional evaluation. If there is a different emotional evaluation, it is used to modify the emotional state from the current situation.

*The Orbitofrontal Cortex (OFC)* is associated with the decision-making process: it is responsible, along with the DLPFC, for seeking and choosing an action [30]. People with damage in this area lose the capacity to make decisions [30]. The DLPFC is associated in the same manner with memory, so the OFC feeds on it to make a decision in any specific situation [19, 30].

We use this area to perform a search and selection of actions from multiple plans generated in collaboration with the DLPFC and the rACC.

*The Amygdala (AMYGDALA)* is believed to collaborate with other structures within the limbic system in assessing the emotional environment [19, 25, 26, 31]. Its internal cores are responsible for the assessments. An entry core is responsible for generating a first emotional evaluation in conjunction with the thalamus. A second entry core receives this first evaluation and subsequently receives a second evaluation from the VLPFC. This second core modifies the emotional meaning of the environment if this is needed. A third core receives the emotional meaning computed in the entry cores and sends it to the rest of the cerebral areas. A fourth core receives the emotional state from the entry cores. It is responsible for maintaining the person's emotional state and sending it to the cerebral areas that require it. This behavior is according to our assumptions and the neuroscientific evidence [31].

The Amygdala in our model is responsible for processing the stimuli, meaning it has the job of emotional assessment within our architecture. It serves to generate an emotional state in collaboration with the motivation to the perceived situation.

*The Insula (INSULA)* has a strong involvement in pain processing [32–35], in addiction studies [36], appetite studies [37], and multimodal sensory integration [33]. Within the work of emotions, its participation is observed both in the generation of the negative affective values of the stimuli and in the generation of emotional behaviors, such as disgust and fear [38, 39]. Within our model, it collaborates on the construction of the emotional state in conjunction with the AMY and the VS, providing affective information.

*The Ventral Striatum (VS)* is involved in motor responses directly related to stimuli perceived with rewards [40]. It is part of the dopaminergic system of the brain, in conjunction with other structures [41]. It is activated during the processing of the reward in both sent signals and the output [40]. Within our model, it collaborates on building the emotional state in conjunction with AMY and INS, providing affective information.

*The Hippocampus (HIPPOCAMPUS)* is associated with the processes of memory. This structure works as an index for past experiences and the emotion felt at the moment they occurred. If there is no previous experience, the Hippocampus is responsible for storing the new experience and its appraisal-emotion is provided by other structures such as the Amygdala [31]. The Hippocampus is associated with the memory of emotions. Damage to this structure can produce memory loss, lack of expressiveness, and even inability to generate emotions [42].

Within the architecture, this structure provides the emotional memory of the perceived environment and is responsible for storing new emotional experiences and retrieving existing ones.

*The rostral Anterior Cingulate Cortex (rACC)* is activated during the exhibition of any emotional response to our behavior. It has no activity during the cognitive processes of the PFC. However, it is believed to be associated with the emotional process. This structure might regulate the emotional behavior on the basis of information provided by the PFC [28].

This structure, in collaboration with the OFC, seeks an appropriate reaction to the situation the environment presents.

*The subgenual Anterior Cingulate Cortex (sgACC)* is associated with emotional behavior. People with depressive or bipolar disorders present more activity in this area than the rest of the population does [27, 28].

In our proposal, this area controls emotional behavior by trying to ignore the current emotion. This objective of controlling emotional behavior depends on the internal emotional state.

*The Sensory Cortex* is the component responsible for encoding perceived environmental stimuli. It refers to the visual, tactile, gustatory, olfactory, and auditory sensory cortex.

*The Motor Cortex* is in charge of executive planning, which generates a frame of execution for the body's reaction to the perceived stimuli.

## 4. Proposal

As established in the introduction, the objective of this article is to endow a virtual creature with a mechanism to compute the appropriate behavior for a specific situation. Our proposal is based on biological requirements previously expressed and ensures that the best reward behavior is computed for the specific situation.

**Figure 2:**
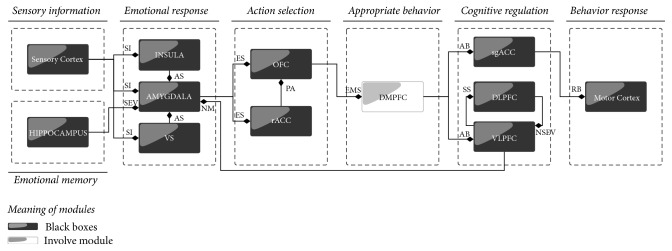
Emotion Regulation Model. A proposed model based on neural evidence and psychological theories (SI = sensory input, AS = affective signal, ES = emotional signal, PA = possible action, EMS = emotional mental state, AB = appropriate behavior, SS = stimulus signal, SEV = stored emotional value, NSEV = new stored emotional value, NM = new meaning, and RB = regulated behavior).

In order to attain our objective, we use neuroscientific evidence regarding emotional regulation. Our proposed model is presented in Figure 2. In this model we can see the neural structures, found at this moment, involved in the process of emotion regulation. In Table 2 we can see the type of information sent by each internal component of the proposed model. We focus on the module we believe is responsible for finding the appropriate behavior (see Involve module in Figure 2). First, we want to describe the functions involved in the proposed model of emotion regulation in order to obtain a global view.

*Sensory Information. *This is the first step, responsible for producing the input signals of the environment to the correct functions of the subsequent steps in the proposed model.

*Emotional Response*. This makes an emotional evaluation of the perceived environment. This step can be executed more than once. It has a direct connection to stimulus perception and to the cognitive region of the brain. We can see this process as a first unconscious response and as a second conscious response (i.e., we assume that the first was already executed) to the environment. As described previously, in this study we deal with the conscious emotional response.

*Action Selection*. In this step an action is selected to be executed in the environment, and it is determined whether an emotional regulation or a change in behavior is needed in order to improve the reward for the specific situation. This step is based on similar previous experiences of the current situation. That is exactly why this process is closely related to the working memory.

*Appropriate Behavior*. In this step, an appropriate behavior is selected for the current situation. To make this selection, a comparison is made between the reward of the action selected and the reward of behaviors selected in similar previous situations. If the process cannot find an appropriate behavior, an option can be selected randomly. However, this is done only if the punishment is not very high. This analysis is achieved in the previous step.

*Cognitive Regulation. *In this step, the system has determined to change the behavior. Thus, different meanings are sought for perceived stimuli in order to try to provoke a change in the emotional response and, consequently, a change in the behavior in the environment. In parallel the suppression mechanism is activated together with the control of physiological behavior, to obtain the appropriate behavior.

*Emotional Memory*. This serves as a place to store and recover the emotional evaluation of the perceived stimulus.

*Behavior Response (This Is the Final Step)*. A physiological behavior response is computed according to the emotional behavior given by the proposed model.

As we can see in the previous descriptions, after activating the emotional regulation mechanism it is necessary to obtain an appropriate behavior. This behavior is computed only if the actual behavior is not appropriate to obtain the specific goal in the environment.

### 4.1. Formal Description of the Evidence

In this work, we propose an adaptive mechanism allowing the appropriate behavior to be computed for autonomous virtual creatures facing a specific situation. In our case (Figure 1), virtual creatures have sensors allowing them to perceive their environment continually and a memory mechanism where they store their experiences. Using these sensors, we want to formally describe the functions necessary for obtaining an appropriate behavior. We do not describe all of functions, because not all of them are necessary to compute the proposed mechanism. In Table 3 we describe the variables used for our proposal.

*Sensory Information* gives the set of stimuli *S* for the subsequent functions in the proposed model. This set is made up of objects and virtual creatures in the environment.

**Figure 3:**
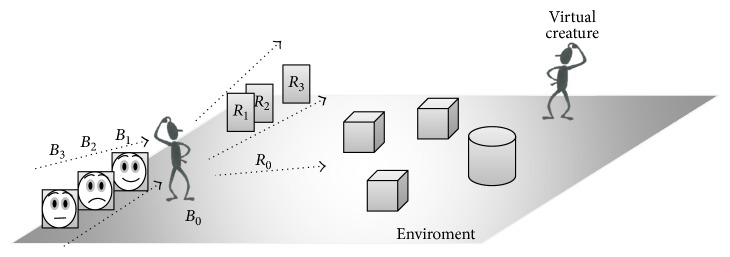
An example of internal emotional action from a virtual creature. Showing possible emotional behaviors from the environment where *B*_1_,…, *B*_*n*_ are possible emotion behaviors and *R*_1_,…, *R*_*n*_ are the possible responses from the environment.

*Emotional response* gives the initial behavior *B*_1_, necessary for the computation of actions. Let *S*_*μ*_ and *S*_*M*_ = {*S*_*M*_*μ*1__, *S*_*M*_*μ*2__,…, *S*_*M*_*μα*__} be a stimulus and its meaning, respectively. Each entry of *S*_*M*_ is another vector of six elements *E* = {*E*_1_, *E*_2_,…, *E*_6_}, where *E*_*x*_ has a real value 0.0 ≤ *E*_*x*_ ≤ 1.0 representing the evaluation of each of the six basic emotions described by Ekman [6]. Let *S*_*μr*_ = *S*_*M*_*μβ*__ be the most relevant evaluation of *S*_*μ*_ (Figure 3). Thus, the initial behavior is computed by *B*_1_ = ∑_*μ*=1_^*n*^*S*_*μr*_.

*Action selection* determines whether the initial behavior *B*_1_ is good for the actual objective in the situation. In the event it is not, it will be necessary to compute another behavior.

**Figure 4:**
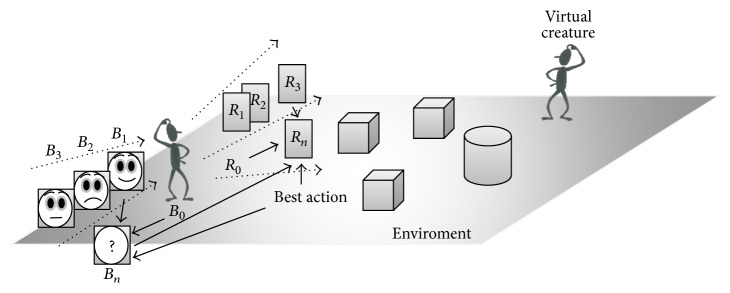
Selection of the rule that best satisfies the conditions of the environment. Showing possible emotional behaviors in response to the environment where *B*_*n*_ are possible emotional behaviors and *R*_*n*_ are the possible responses from the environment.

*Appropriate behavior* tries to generate an appropriate behavior taking into account the initial behavior *B*_1_ and the actual situation ST (Figure 4). That is, we know that the computed behavior *B*_1_ is not suitable to reach the final objective; however, we obtain useful information as the starting point to compute the appropriate behavior.

## 5. Implementation of the Appropriate Behavior Function

**Figure 5:**
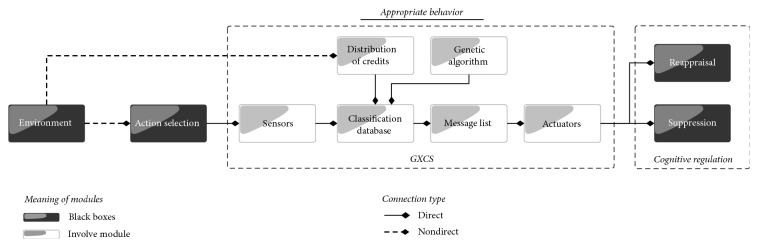
General process of GXCS. Showing a general process of GXCS with inputs and outputs.

In order to compute the* appropriate behavior* function, we choose to work with a learning classifier system (LCS), because this sort of tool allows for the exhaustive search, with the possibility of stopping the search in order to explore local solutions while working to find global solutions. The learning classifier system or LCS was proposed initially by Holland [43, 44]. This LCS combines genetic algorithms and machine-learning techniques. In this article, we use the LCS called GXCS [45] (Figure 5). This LCS was developed by searching its applications specifically to solve problems in the area of virtual agents. However, it can be applied in different areas. The GXCS goes beyond the representation of binary rules and makes it possible to use any kind of data. In our case, it allows us to use the most natural and appropriate data to represent the environment.

**Figure 6:**
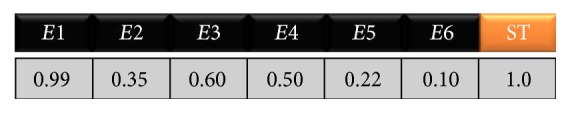
A sample of a rule's structure for LCS. Showing a syntax rule where *E*1, *E*2,…, *E*6 are emotions and ST is the current situation.

The GXCS provides a way to assign a behavior to a virtual creature based on the characteristics of the current setting. That is, the GXCS uses the similarity between the current setting and previous experiences (settings) to compute the virtual creature's optimal behavior for the current setting. In order to use this GXCS, whose behavior is explained in [45], first we need to define the vector rule used for this purpose. The basic emotions proposed by Ekman [6] and the identification of a situation are embedded in the vector. That is, our vector rule has 7 entries, six for the basic emotion given by *E*_1_, *E*_2_,…, *E*_6_ and the last used to identify the situation corresponding to that emotional evaluation. Each of the entries has real values bounded by 0 and 1 (Figure 6).

The internal behavior of the six components of our GXCS for this study (see Figure 5) is described extensively below.

*Sensors *form an interface, fed by the previous action selection function, in which the aforementioned rule of 7 inputs is predefined {*E*_1_, *E*_2_, *E*_3_, *E*_4_, *E*_5_, *E*_6_, ST} (Figure 6).

*Classification database* represents the knowledge base of the system. It consists of a population of* n* rules of the type condition : action, where the condition is provided by the sensors and the action is produced by the genetic algorithm.

*Genetic algorithm* is responsible for generating new actions based on the best evaluated rules existing within the knowledge base. If no rule exists, actions are randomly formulated.

*Distribution of credits* is the function in charge of evaluating the condition : action rules existing within the classification database. This function requires feedback from the environment, which helps determine the impact of the rule used in the current situation.

**Figure 7:**
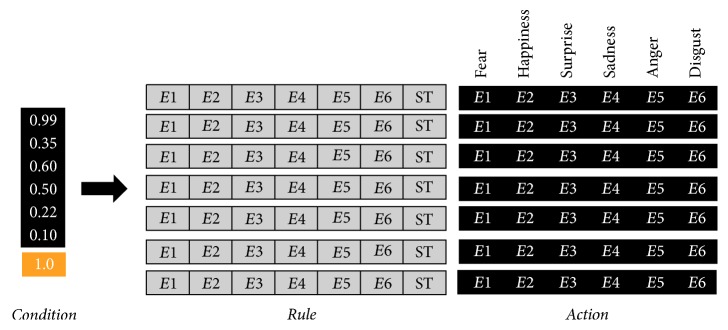
A sample of a rule's database for GXCS. Showing a syntax rule where *E*1, *E*2,…, *E*6 are emotions and ST is the current situation.

*Message list* is the set of condition : action rules retrieved from the classification database associated with the input rule provided by the sensors. See Figure 7.

*Actuators* are the output interface composed of effectors, responsible for expressing the action determined by the chosen rule of the message list. The actions consist of 6 exits {*E*_1_, *E*_2_, *E*_3_, *E*_4_, *E*_5_, *E*_6_} associated again with Ekman's basic emotions [6]. Each of the exits has real values bounded by 0 and 1.

**Figure 8:**
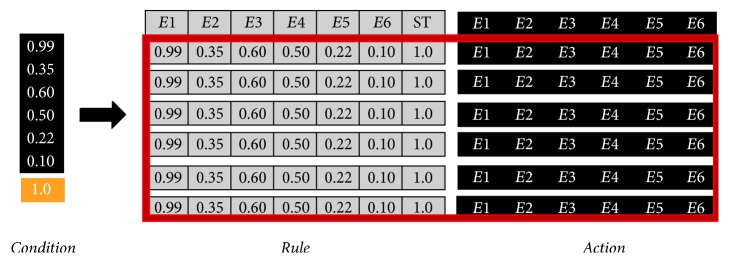
A sample of generated rules for GXCS. Showing rules generated by an action with 6 components.

This implementation also uses unsupervised reinforcement learning. On the basis of the environment, a certain expected behavior of the virtual creature is defined; if the behavior that the virtual creature exhibits comes close to the environmentally predefined behavior, the rule formulator of this behavior receives a high reward. The way to provide such a reward is simple: first define an emotion (joy, fear, sadness, disgust, or anger) on the basis of the environment, and then the virtual creature should seek to express that emotion. If it does, the intensity of the emotion will determine the recombination obtained, which will be provided to the GXCS in order to evaluate the generating rule; otherwise, the rule will be punished by being assigned values of 0. For example, let us say the expected behavior is predicted in the environment by the array {0.0, 0.0, 1.0, 0.0, 0.0, 0.0, 0.0}, the first 6 fields being expected emotional intensities and the last one, an identifier of the situation. On the other hand, the virtual creature expresses a behavior given by the array {0.2, 0.4, 0.6, 1.0, 0.8, 0.0}, where all fields are emotional intensities. The generating rule will be evaluated with 0.6 of reward, since it is approaching the emotional value specifically descaled in the emotion of field three. Initially, the rules are generated randomly and they feed the GXCS. The rules are graded from 0.0 to 1.0, where 0.0 is a very poor response and 1.0 is a high emotional intensity. The GXCS offers the possibility to experiment or take the best option. This means that we can choose to use one existent rule or use this information to generate a new rule hoping it will be better suited to the environment's stimuli. The number of generated rules is given by the number of components of the action vector (Figure 8). We maintain the experimentation until we achieve a rating of 0.8 or higher on any rule within a new situation. A rule is associated with a behavior, and a highly evaluated rule is considered an appropriate behavior. This type of rule is functional and it is stored. The nonfunctional rules or rules with a low evaluation are also stored, because they may be useful in a similar situation in the future.

### 5.1. Case Study: Unfamiliar Situations

In unfamiliar situations, the GXCS at first does not have a specific number of rules to converge on a specific emotional valuation. The objective, within this case study, is to get the virtual creature to converge on a certain response behavior. We do this by defining a specific behavior in the environment and providing reward values, as explained above. The case study uses three different emotions in each simulation; that means that the virtual creature converges on three different emotional behaviors in a single situation. The GXCS is not stopped, so there are evaluated rules belonging to other emotional responses. The simulation runs 20 times to determine which number of rules converges on the appropriate behavior and its intensity.

#### 5.1.1. Results

**Figure 9:**
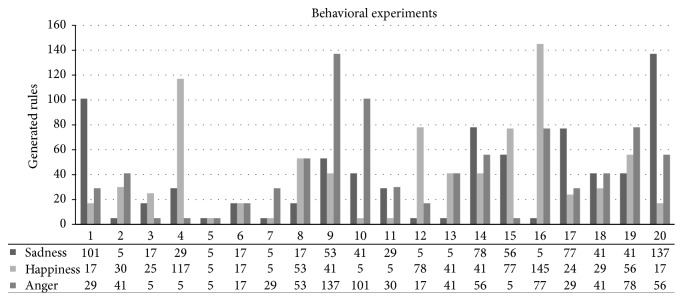
Graph of search behavior showing results from the first experiment in three columns. Each column is one emotion; the numbers are the rules generated in the experiment. The time taken for each experiment was less than one second.

In this experiment, where the virtual creature does not have previous experiences, the results are shown in the graph of search behavior (Figure 9). In this case, the number of rules generated by the classifier ranges between 5 and 145. In 20 tries, the average was 50 rules. In all cases, the behavior that was sought was a specific emotion (as defined in the graph) with a rating of 0.8 or higher. In all the events a rule was generated containing the emotional value within the first 20 rules.

### 5.2. Case Study: Familiar Situations

In a familiar situation, the initial set of rules allows the GXCS to look for appropriate behaviors to respond to an unknown situation in the environment. The continuous environment evaluation enables the system to converge, if possible, towards an appropriate behavior. The GXCS associates the unknown situation with situations that are already partially known; that means there are a few differences in the situation and the emotional mental state of the creature (condition of GXCS) is slightly changed. The classifier relates the rules of the previous situations in order to propose an approximately appropriate behavior. This experiment consists of five simulations with five different settings each; each setting changes a little in order to show the number of rules generated in familiar situations.

#### 5.2.1. Results

**Figure 10:**
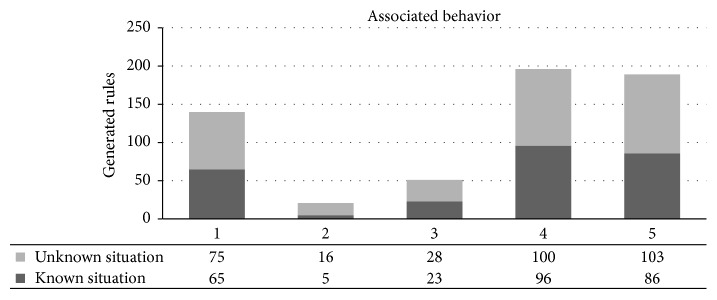
Graph of association of behavior. Showing a result of the second experiment, with two columns—unknown situations and known situations—for each experiment using the same LCS. The difference between the unknown and known situation was less than 15 new rules.

The graph (Figure 10) shows the number of rules that were required to generate the appropriate behavior for a specific setting. The number of rules generated for appropriate behavior in each of the experiments varied in the range of 5–20, with an average of 7 rules over 5 experiments. The generation time of the new rules is between 1 and 4 seconds for an unknown but similar setting (modified setting). When the classifier receives a setting similar to one it has already learned, it proceeds as follows to compute appropriate behavior: it first calculates the similarity with the known setting and then calculates the appropriate behavior using the behavior associated with an initial behavior from the setting with the closest similarity.

## 6. Discussion

**Figure 11:**
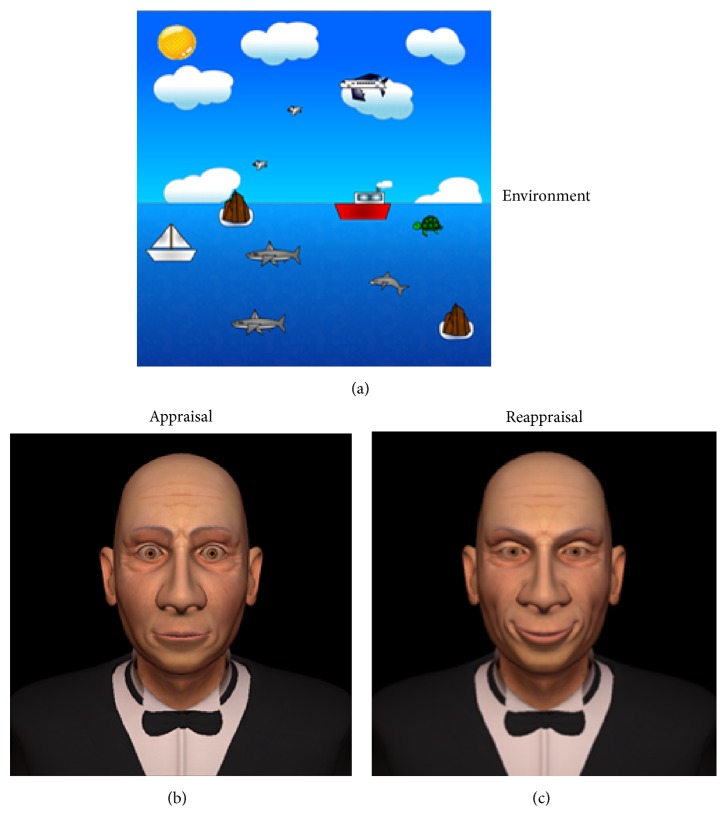
Alfred sample. This picture shows how the model of emotional regulation works. Part (a) shows the environment with difference evaluations stored in the memory; part (b) shows Alfred with an expression of fear, which is the first emotion. Applying the LCS to obtain the appropriate behavior, we have a visible decrease of emotion seen in Alfred in part (c). This result is obtained from the appropriate behavior given by LCS and multiple evaluations of emotional objects in the setting using an emotion regulation architecture.

As we established previously, the objective of this research is to propose a mechanism whereby a virtual creature can autonomously calculate appropriate behavior for a specific situation (Figure 11). Our proposal is to create a mechanism for the virtual creature to be able to choose an appropriate behavior in a specific situation. The mechanism is based on neuroscientific and psychological evidence. From neuroscience, we obtain some of the cerebral regions involved in this process, the functions they achieve, and the flow of the information.

In the proposed model, not all of the brain structures described in the state of the art concerning emotion regulation are involved [13, 26]. This is because there is currently no consensus on which structures actually contribute. For this reason, in our proposed model we consider only structures for which there is consensus. These structures are described in Section 4. In other words, on the basis of neuroscientific evidence we propose that the DMPC is the structure responsible for computing behavior that is appropriate to a specific situation (see Figure 2). In addition, there is also evidence that some brain structures are involved in different ways in the tasks of different cognitive functions. In this study, we focus on the functions of the brain structures that contribute to the calculation of a behavior for a situation that requires emotion regulation.

One might think that the search for appropriate behavior would only favor the so-called “social cognitive emotion regulation” [13]. However, there is evidence that, in specific cases, for example, survival situations where it is necessary to control emergent behavior (fear) to avoid a predator, emotion regulation is required in addition to appropriate behavior. This is why we propose in our model the activation of the DMPFC structure in both social regulation and self-regulation [13–15] in order to calculate appropriate behavior. Along the same lines, as mentioned previously in the introduction, having the appropriate behavior is necessary for the proper functioning of cognitive emotion regulation techniques (suppression and reevaluation); a more detailed description of this is described in [3].

Regarding the implementation and results of the case studies presented, we can underline first the use of an LCS named GXCS proposed by M. A. Ramos and F. Ramos [45], which allowed us to use a structure that summarizes the six basic emotions defined by Ekman [6] as well as a field that associates the emotions with a specific situation (which provokes the emotional state, e.g., desert, sea, and field). This structure is simplistic and overlooks multiple aspects of emotion regulation, such as social interaction or risk situations, but it is a first approach to explore the functionality of an LCS in learning appropriate behaviors for creatures subjected to situations requiring emotion regulation.

Second, behaviors simply refer to Ekman's 6 basic emotions and are the virtual creature's calculated and displayed behavior in each experiment. This behavior receives a high evaluation if it approaches the appropriate behavior for the situation and a low evaluation in the opposite case.

Third, from the results we must emphasize that, in the unfamiliar case study, the system does not know the appropriate behavior and only has the creature's internal state and a specific context. In this case, the first rules are randomly generated to initiate the evaluation of behaviors; each of these rules is evaluated within the system using the same situation, thus setting off an iteration using the rules that had better performance in the specific situation. The iteration runs until an appropriate behavior is obtained.

In Figure 9 we can see a summary of the results obtained: the number of rules formulated in each situation. Each situation is shown by means of 3 columns, which represent different emotions. We can also observe that there are considerably high numbers of generated rules, reaching 145 rules evaluated. This was expected since technically the answers given by the virtual creature are almost random, but in all situations it was possible to get to the behavior that was appropriate to the situation presented.

With respect to the family case study, the system already had a priori rules associated with the same context, but with different internal emotional states. In this case, the results obtained are considerably better compared to the results of the unfamiliar case study, approximately 15 more rules to identify unknown situations but with similarities. This case shows the performance of the LCS when it has a priori knowledge about the situations presented to it.

In spite of the very good results we have obtained, there is still work to do, primarily the optimizations to improve the performance of our proposal in both time and quality. For example, in the implementation, we define explicit values for the emotions used for seeking an appropriate behavior. This knowledge can improve efficiency, making the GXCS discriminate useless elements during the process of seeking a behavior for a situation. Another possible area of improvement is the evaluation method, which can be improved by reducing the set of rules evaluated to those that approach the appropriate behavior. This is possible because we have sought the value of the rule. Another possible improvement we are currently working on is the selection of rules within the classifier to consider. We want the learning classifier system to consider the evaluation of a larger number of related rules in order to reduce the rule generation process, which is quite costly.

Finally, we have proposed an automatic way of calculating appropriate behavior in situations requiring emotion regulation. Our proposal is based on neuroscientific evidence. This proposal is novel, since it is not contemplated by proposals of the state of the art, which are oriented more towards the correct operation of the cognitive regulation techniques or towards the identification of the context and the current situation.

### 6.1. Conclusions

Although our proposed model of emotion regulation cannot be considered complete, it contains the necessary foundations for the search for appropriate behavior in a situation that requires emotion regulation. This behavior, as we have already mentioned, is the fundamental component to carry out emotional cognitive regulation. From the implementation presented we can observe that the proposal is appropriate, because the virtual creature is able to calculate an appropriate behavior from scratch for a situation that requires emotion regulation when the creature does not have previous experiences and in an efficient way when it does have previous experiences for the situation it is facing. In the same way, we can conclude that the use of a GXCS classifier to carry out the learning of the appropriate behavior is viable and favors us in similar situations by giving us quick answers. On the other hand, we can also conclude that it is possible to improve the efficiency of the system by making adjustments, for example, by improving the generation of GXCS rules to accelerate the calculation of appropriate behaviors.

## Figures and Tables

**Table 1:** Description of areas. General architecture structures for emotion regulation, inputs, and outputs.

Structure	Input	Output
AMYGDALA	Frame with preprocessed and processed information.	Frame with emotional information.
HIPPOCAMPUS	Requests.	Information retrieved from the emotional memory.
INSULA	Sensory information.	Affective information oriented towards pain.
VS	Sensory information.	Affective information oriented towards pleasure.
rACC	Frame with processed information and emotional state.	Possible actions.
OFC	Possible actions and emotional state.	The frame of selected action.
DMPFC	The frame of action and information from memory.	Emotional state and appropriate emotional behavior.
DLPFC	Requests.	Information retrieved from working memory.
VLPFC	Required emotional state.	The cognitive-emotional change closest to what is required.
sgACC	Emotional behavior and emotional state.	General behavior.
Sensory Cortex	Environmental information.	Sensory information.
Motor Cortex	General behavior.	Execution frame.

**Table 2:** Informal description of input and output of a proposed model.

Label	Meaning	Description
SI	Sensory input	Sensory values from the environment
AS	Affective signal	Affective values computed by the system
ES	Emotional signal	Emotional values associated with emotions
PA	Possible action	Possible action to run in the environment
EMS	Emotional mental state	Current emotional state of the virtual creature
AB	Appropriate behavior	Appropriate emotional behavior to be computed
SS	Stimulus signal	Sign of stimuli perceived in the environment
SEV	Stored emotional value	Emotional values stored from past experiences
NSEV	New stored emotional value	New emotional value to be stored for a stimulus
NM	New meaning	New emotional meaning of the situation
RB	Regulated behavior	Regulated behavior to be expressed

**Table 3:** Variables used in our proposal and their meaning.

Variable	Meaning
*S*	Stimuli set
*S*_*μ*_	Stimulus *μ*
*S*_*M*_	Stimulus meaning
*E*1, *E*2,…, *E*6	Six basic emotions described by Ekman 1994 [6]
*Bz*	Behavior *z*
*μ*, *x*, *n*, *z*	Integers used as indices

## References

[1] Gross J. J. (1998). The emerging field of emotion regulation: an integrative review. *Review of General Psychology*.

[2] Scherer K. R., Schorr A., Johnstone T. (2001). *Appraisal Processes in Emotion: Theory, Methods, Research. Series in Affective Science*.

[3] Rosales J. H., Jaime K., Ramos F. (2013). *An Emotional Regulation Model with Memories for Virtual Agents*.

[4] Gross J. J. (2002). Emotion regulation: affective, cognitive, and social consequences. *Psychophysiology*.

[5] Thompson R. A. (1988). Emotion and self-regulation.. *Nebraska Symposium on Motivation*.

[6] Ekman P. A. (1994). *The nature of emotion: fundamental questions*.

[7] Laird J. E., of Technology M. I. (2012). The Soar Cognitive Architecture. *The Soar Cognitive Architecture. (M. I. of Technology*.

[8] Rodríguez L.-F., Ramos F., Wang Y. Cognitive Computational Model of Emotions.

[9] Vernon D. A. (2011). *A Roadmap for Cognitive Development in Humanoid Robots*.

[10] Breazeal C. (2003). Emotion and sociable humanoid robots. *International Journal of Human-Computer Studies*.

[11] Breazeal C., Scassellati B. (2000). Infant-like social interactions between a robot and a human caregiver. *Adaptive Behavior*.

[12] Breazeal C. (1998). Early experiments using motivations to regulate human-robot interaction. *AAAI*.

[13] Xie X., Mulej Bratec S., Schmid G. (2016). How do you make me feel better? Social cognitive emotion regulation and the default mode network. *NeuroImage*.

[14] Cooper J. C., Furey T. A. (2012). Dorsomedial prefrontal cortex mediates rapid evaluations predicting the outcome of romantic interactions. *The Journal of Neuroscience*.

[15] Grinband J., Savitskaya J., Wager T. D., Teichert T., Ferrera V. P., Hirsch J. (2011). The dorsal medial frontal cortex is sensitive to time on task, not response conflict or error likelihood. *NeuroImage*.

[16] Campbell-Sills L., Simmons A. N., Lovero K. L., Rochlin A. A., Paulus M. P., Stein M. B. (2011). Functioning of neural systems supporting emotion regulation in anxiety-prone individuals. *NeuroImage*.

[17] Banks S. J., Eddy K. T., Angstadt M., Nathan P. J., Luan Phan K. (2007). Amygdala-frontal connectivity during emotion regulation. *Social Cognitive and Affective Neuroscience*.

[18] Phan K. L., Fitzgerald D. A., Nathan P. J., Moore G. J., Uhde T. W., Tancer M. E. (2005). Neural substrates for voluntary suppression of negative affect: a functional magnetic resonance imaging study. *Biological Psychiatry*.

[19] Miller L. A., Taber K. H., Gabbard G. O., Hurley R. A. (2005). Neural underpinnings of fear and its modulation: implications for anxiety disorders. *J Neuropsychiatry ClinNeurosci*.

[20] Hariri A. R., Mattay V. S., Tessitore A., Fera F., Weinberger D. R. (2003). Neocortical modulation of the amygdala response to fearful stimuli. *Biological Psychiatry*.

[21] Davidson R. J., Putnam K. M., Larson C. L. (2000). Dysfunction in the neural circuitry of emotion regulation—a possible prelude to violence. *Science*.

[22] Bennett M. R. (2011). The prefrontal-limbic network in depression: a core pathology of synapse regression. *Progress in Neurobiology*.

[23] Barbas H. (2009). Prefrontal cortex: structure and anatomy. *Encyclopedia of Neuroscience*.

[24] Fuster J. M. (2008). *The Prefrontal Cortex*.

[25] Kandel E. R. (2000). *Principles of Neural Science*.

[26] Barrett L. F., Satpute A. (2013). Large-scale brain networks in affective and social neuroscience: Towards an integrative functional architecture of the brain. *Current Opinion in Neurobiology*.

[27] Drevets W. C., Savitz J., Trimble M. (2008). The subgenual anterior cingulate cortex in mood disorders. *CNS Spectrums*.

[28] Bush G., Luu P., Posner M. I. (2000). Cognitive and emotional influences in anterior cingulate cortex. *Trends in Cognitive Sciences*.

[29] Petrides M. (2000). The role of the mid-dorsolateral prefrontal cortex in working memory. *Executive Control and the Frontal Lobe: Current Issues*.

[30] Wallis J. D. (2007). Orbitofrontal cortex and its contribution to decision-making. *Annual Review of Neuroscience*.

[31] Whalen P. J., Phelps E. A. (2009). *The Human Amygdala*.

[32] Hashmi J. A., Baliki M. N., Huang L. (2013). Shape shifting pain: chronification of back pain shifts brain representation from nociceptive to emotional circuits. *Brain*.

[33] Treede R.-D., Apkarian A. V., llan A. K., Basbaum I. (2008). *5.45 - nociceptive processing in the cerebral cortex*.

[34] Apkarian A. V., Bushnell M. C., Treede R.-D., Zubieta J.-K. (2005). Human brain mechanisms of pain perception and regulation in health and disease. *European Journal of Pain*.

[35] Legrain V., Iannetti G. D., Plaghki L., Mouraux A. (2011). The pain matrix reloaded: a salience detection system for the body. *Progress in Neurobiology*.

[36] Naqvi N. H., Rudrauf D., Damasio H., Bechara A. (2007). Damage to the insula disrupts addiction to cigarette smoking. *Science*.

[37] Dagher A. (2012). Functional brain imaging of appetite. *Trends in Endocrinology & Metabolism*.

[38] Arce E., Simmons A. N., Lovero K. L., Stein M. B., Paulus M. P. (2008). Escitalopram effects on insula and amygdala BOLD activation during emotional processing. *Psychopharmacology*.

[39] Stein M. B., Simmons A. N., Feinstein J. S., Paulus M. P. (2007). Increased amygdala and insula activation during emotion processing in anxiety-prone subjects. *The American Journal of Psychiatry*.

[40] Palminteri S., Justo D., Jauffret C. (2012). Critical Roles for Anterior Insula and Dorsal Striatum in Punishment-Based Avoidance Learning. *Neuron*.

[41] Smith K. S., Berridge K. C., Aldridge J. W. (2011). Disentangling pleasure from incentive salience and learning signals in brain reward circuitry. *Proceedings of the National Acadamy of Sciences of the United States of America*.

[42] Andersen P. A. (2007). *The Hippocampus Book*.

[43] Holland J. H. Properties of the bucket brigade.

[44] Holland J. H. (1976). Adaptation. *Progress in Theoretical Biology*.

[45] Ramos M. A., Ramos F. Autonomous agents and anticipative systems.

